# Multi-Responsive Amphiphilic Hyperbranched Poly[(2-dimethyl aminoethyl methacrylate)-co-(benzyl methacrylate)]copolymers: Self-Assembly and Curcumin Encapsulation in Aqueous Media

**DOI:** 10.3390/ma18030513

**Published:** 2025-01-23

**Authors:** Foteini Ginosati, Dimitrios Vagenas, Angelica Maria Gerardos, Stergios Pispas

**Affiliations:** Theoretical and Physical Chemistry Institute, National Hellenic Research Foundation, 48 Vassileos Constantinou Ave., 11635 Athens, Greece; fotini.gin97@gmail.com (F.G.); dimitrisv98@gmail.com (D.V.); amgerar@eie.gr (A.M.G.)

**Keywords:** amphiphilic hyperbranched statistical copolymers, RAFT polymerization, self-assembly, light scattering, encapsulation, curcumin

## Abstract

In this study, we report the synthesis of amphiphilic hyperbranched poly[(2-dimethylaminoethyl methacrylate)-co-(benzyl methacrylate)] statistical copolymers with two different stoichiometric compositions using the reversible addition–fragmentation chain transfer polymerization (RAFT) technique. The selection of monomers was made to incorporate a pH and thermoresponsive polyelectrolyte (DMAEMA) component and a hydrophobic component (BzMA) to achieve amphiphilicity and study the effects of architecture and environmental factors on the behavior of the novel branched copolymers. Molecular characterization was performed through size exclusion chromatography (SEC) and spectroscopic characterization techniques (^1^H-NMR and FT-IR). The self-assembly behavior of the hyperbranched copolymers in aqueous media, in response to variations in pH, temperature, and ionic strength, was studied using dynamic light scattering (DLS), electrophoretic light scattering (ELS), and fluorescence spectroscopy (FS). Finally, the efficacy of the two novel copolymers to encapsulate curcumin (CUR), a hydrophobic, polyphenolic drug with proven anti-inflammatory and fluorescence properties, was established. Its encapsulation was evaluated through DLS, UV–Vis, and fluorescence measurements, investigating the change of hydrodynamic radius of the produced mixed copolymer–CUR nanoparticles in each case and their fluorescence emission properties.

## 1. Introduction

Hyperbranched polymers (HBPs) have gained significant interest due to their unique three-dimensional structure, which provides several advantages over traditional linear polymers. As research on HBPs has expanded rapidly, the focus has shifted toward their synthesis, self-assembly, and diverse applications. Notably, their key features, such as low viscosity, high solubility, and a high density of functional end groups, make them versatile for drug delivery, tissue engineering, and advanced material design. In comparison to linear polymers, HBPs demonstrate lower molecular entanglement and higher reactivity, along with a greater ability to encapsulate both hydrophilic and hydrophobic molecules, all owing to their irregular, branched, and nearly globular architecture. Furthermore, their self-assembly behavior plays a crucial role in their capability to form stable structures, making them highly effective in targeted drug delivery and other biomedical applications [1,2].

Extending these benefits, amphiphilic hyperbranched polymers (AHPs) demonstrate unique capabilities that make them particularly suited as nanovectors. Due to their branched structure and multiple binding sites, AHPs exhibit the ability to encapsulate small molecule “guests”, including organic dyes. The encapsulation efficiency of AHPs depends on factors such as the molecular weight, degree of branching (DB), shell density, and the polarity gradient between the core and shell [3]. Stimuli-responsive HBPs can react to environmental changes such as pH, temperature, or other external stimuli by undergoing significant alterations in shape, volume, or phase state. This responsiveness allows HBPs to release drugs in a controlled manner at specific sites, such as tumor tissues, where conditions like pH differ from the rest of the body [4]. pH-responsive HBPs, in particular, have shown promise in cancer therapy by enabling targeted release and uptake in acidic tumor microenvironments [5]. Furthermore, thermoresponsive HBPs can show transitions in response to temperature changes, which is advantageous for bioapplications like controlled drug release and solid–liquid separation processes.

Multi-stimuli-responsive HBPs, which can react to multiple triggers, offer enhanced versatility in designing smart materials for biomedicine and biotechnology [6]. Stimuli-responsive polymeric systems have seen significant advancements in their synthesis through controlled radical polymerization (CRP) techniques, such as reversible addition–fragmentation chain transfer (RAFT) polymerization. RAFT polymerization employs chain transfer agents (CTAs) to regulate the growth of polymer chains through a reversible activation–deactivation mechanism. This process allows for precise control over molecular weight, composition, and architecture, making it particularly effective for synthesizing complex polymer structures. In the context of hyperbranched polymers, RAFT facilitates better control over branching points and chain length, effectively minimizing gelation [7,8,9]. Poly(2-(dimethylamino)ethyl methacrylate) (PDMAEMA) is a polymer recognized for its dual responsiveness to pH and temperature changes. The pH responsiveness is attributed to the protonation and deprotonation of its tertiary amine groups, causing a shift between hydrophilic and hydrophobic states [10]. PDMAEMA also exhibits thermoresponsive behavior with a lower critical solution temperature (LCST) typically ranging between 32–50 °C, influenced by factors like molecular weight and solution conditions [11,12]. The ionic strength of the solution further impacts the properties of crosslinked/branched PDMAEMA. The dual-responsive nature of PDMAEMA has led to its extensive use in a range of applications, including gene and drug delivery systems, antibacterial agents, hemostatic materials, filtration techniques, coatings, and biomedical engineering [13,14].

In the present work, we report on the synthesis of novel poly[(2-dimethylamino ethyl methacrylate)-co-(benzyl methacrylate)], P(DMAEMA-co-BzMA), hyperbranched copolymers via RAFT polymerization. The polymers were synthesized in two different ratios of DMAEMA:BzMA monomeric units, where one ratio was targeted to 80% DMAEMA:20% BzMA (HB1), and the other was targeted to 60% DMAEMA:40% BzMA (HB2), with ethylene glycol dimethacrylate (EGMA) used as the branching agent. DMAEMA was used as the dual-responsive hydrophilic component of the copolymers, while benzyl methacrylate was incorporated to impart the amphiphilic character of the copolymers due to its hydrophobic nature [15,16]. We investigated the self-organization behavior of the copolymers in aqueous media in response to various physicochemical stimuli including pH, temperature, and ionic strength, through light scattering techniques and fluorescence spectroscopy. Lastly, we tested the ability of the hyperbranched copolymers to encapsulate curcumin—a hydrophobic phenolic compound known for its antioxidant, anti-inflammatory, and antitumor properties and its low toxicity. It is also utilized in bioimaging as a fluorescent probe, due to its non-toxic and photostable characteristics [17,18,19]. The stability of the loaded nanocarriers was examined and the fluorescence characteristics of these carriers were evaluated.

## 2. Materials and Methods

### 2.1. Materials

The monomers 2-(dimethylamino)ethyl methacrylate (DMAEMA) and benzyl methacrylate (BzMA), and the branching agent ethylene glycol dimethacrylate (EGDMA, 98%, difunctional monomer), were obtained from Sigma-Aldrich (Athens, Greece). DMAEMA, EGDMA, and BzMA were purified before polymerization by passing them through a column packed with inhibitor-removing resin (311,332, Sigma-Aldrich). The radical initiator, 2,2-azobis(isobutyronitrile) (AIBN), was recrystallized from methanol before use. The solvent, 1,4-dioxane (≥99.8% pure, Sigma-Aldrich), was dried using molecular sieves, while acetone, n-hexane (≥97%, Aldrich), chain transfer agent, 4-cyano-4-(phenylcarbonothioylthio) pentanoic acid (CPAD), fetal bovine serum (FBS), and other reagents were used as received from Sigma-Aldrich.

### 2.2. Synthesis of P(DMAEMA-co-BzMA) Hyperbranched Copolymers

For the synthesis of hyperbranched P(DMAEMA-co-BzMA) copolymers via a one-pot RAFT polymerization, CPAD was utilized as the chain transfer agent (CTA), AIBN as the radical initiator, and 1,4-dioxane as the reaction solvent. The synthesis of a representative hyperbranched copolymer (HB1) is detailed as follows:In a 50 mL single-necked round-bottom flask equipped with a magnetic stirrer, DMAEMA (2.4 g, 15.2 mmol), BzMA (0.6 g, 3.4 mmol), ethylene glycol dimethacrylate (EGDMA) (0.143 g, 0.72 mmol), CPAD (0.168 g, 0.6 mmol), AIBN (0.049 g, 0.3 mmol), and 1,4-dioxane (to a total volume of 10 mL) were combined. The molar ratio of CPAD to AIBN was adjusted to 2:1, targeting a molecular weight of 5000 g/mol for a linear copolymer case. The resulting mixture was homogenized under stirring after sealing the flask with a rubber septum and subsequently degassed by nitrogen gas flowthrough syringes for 20 min. Then, the reaction mixture was heated in an oil bath at 70 °C with continuous stirring for 24 h, allowing the polymerization to proceed. Upon completion, the flask was cooled to −20 °C for 20 min and then exposed to air to terminate the reaction. The crude reaction mixture was precipitated in a large excess of n-hexane to remove unreacted monomers and other byproducts. The resulting hyperbranched copolymer was collected and dried in a vacuum oven to obtain the final product.

### 2.3. Self-Assembly of P(DMAEMA-co-BzMA) in Aqueous Media

Stock solutions of hyperbranched copolymers were prepared by using the nanoprecipitation technique. Specifically, the hyperbranched copolymers were dissolved in acetone and rapidly injected in water under stirring using a syringe, promoting self-assembly of the amphiphilic copolymers. The mixture was then heated above the boiling point of the organic solvent (65 °C) on a hot plate, facilitating the gradual evaporation of the organic phase and resulting in nanoparticle formation in the aqueous medium. Stock solutions were prepared for the two copolymers (HB1 and HB2), at a fixed polymer concentration of 10^−3^ g/mL and a pH of 7. These solutions were allowed to equilibrate overnight before further analysis. To explore the potential effects of pH on the assembly behavior of the copolymers, the aqueous solutions were prepared at pH values of 3 and 10. The desired pH levels were achieved by carefully adjusting the solutions with 0.1 M HCl for acidic conditions and 0.1 M NaOH for basic conditions. The solutions were filtered through hydrophilic PVDF filters with a pore size of 0.45 μm prior to conducting dynamic light scattering measurements.

### 2.4. Ionic Strength Study

Polyelectrolytes with ionic groups often exhibit sensitivity to ionic strength, influencing properties such as nanoparticle size and solubility. To investigate this effect, NaCl (1 M) was incrementally added to aqueous solutions to achieve final salt concentrations of 0.1 M, 0.33 M, and 0.5 M. These modifications were monitored for changes in scattering intensity and hydrodynamic radius (R_h_) using dynamic light scattering (DLS) measurements, performed at a 90° angle.

### 2.5. Curcumin Encapsulation into P(DMAEMA-co-BzMA) Aggregates

The procedure for encapsulating curcumin (CUR) into hyperbranched copolymer nanostructures is outlined below. Two stock solutions were prepared: one containing the copolymer dissolved in acetone and another with curcumin in acetone. In detail, to prepare HB1–10% *w*/*w* CUR (HB1 CUR10), 0.01 g of copolymer and 0.00010 g of curcumin were dissolved in acetone. Then, the copolymer–curcumin organic solution was rapidly injected in water under stirring using a syringe. The organic solvent was removed by heating at 65 °C. The curcumin-loaded nanostructures were characterized using FTIR spectroscopy and DLS measurements (at a 90° angle) to confirm their formation, size, and stability. Measurements were conducted on the preparation day and every two days after that for up to 12 days. Additionally, UV–Vis spectroscopy was employed to determine the maximum absorbance of the curcumin-loaded hyperbranched copolymers.

### 2.6. FBS Interaction Study

To investigate potential interactions between the hyperbranched copolymers and blood proteins, the copolymers were combined with fetal bovine serum (FBS). Specifically, aqueous copolymer solutions were mixed with an FBS:WFI (water for injection) solution at a 1:1 *v/v* ratio (50% *v/v* FBS–50% *v/v* WFI). Dynamic light scattering (DLS) measurements were performed one hour after sample preparation, and the results were compared to those obtained for pure FBS. Prior to analysis, all samples were filtered using 0.45 μm pore size hydrophilic PVDF filters.

### 2.7. Characterization Methods

#### 2.7.1. Size Exclusion Chromatography (SEC)

The molecular weights and molecular weight distributions of the hyperbranched copolymers were analyzed using SEC. The setup included a Waters 1515 isocratic pump, a set of three μ-Styragel mixed-composition separation columns (with a pore range of 10^2^–10^6^ Å), and a Waters 2414 refractive index detector maintained at 40 °C, with data processed using Breeze software. Tetrahydrofuran (THF) containing 5% *v/v* triethylamine was employed as the mobile phase at a flow rate of 1.0 mL/min at 30 °C. The system was calibrated with linear polystyrene standards featuring narrow molecular weight distributions and weight-average molecular weights ranging from 1200 to 920,000 g/mol.

#### 2.7.2. ^1^H-NMR Spectroscopy

Nuclear Magnetic Resonance (NMR) spectroscopy was performed using a Varian 300 spectrometer operating at 300 MHz. Spectra acquisition was carried out with the VNMR software (2.2C, Varian, Palo Alto, CA, USA), while the MestReNova software (version 6.0.2, Mestrelab Solutions, Bajo, Spain) was utilized for data analysis. The samples were prepared in deuterated acetone-d_6_ at a polymer concentration of approximately 10 mg/mL. Chemical shifts were reported in parts per million (ppm), with tetramethylsilane serving as the internal reference.

#### 2.7.3. Fourier Transform Infrared Spectroscopy (FT-IR)

The Attenuated Total Reflectance Fourier Transform Infrared (ATR-FTIR) spectra of the hyperbranched copolymer samples were recorded using a Bruker Equinox 55 spectrometer (Bruker Optics GmbH, Billerica, MA, USA) equipped with a single-bounce ATR diamond accessory (Dura-Samp1IR II, SensIR Technologies, Danbury, CT, USA). Each spectrum was obtained by averaging 30 scans at a resolution of 2 cm^−1^. Polymers were measured in solid form using a press, while for the drug-loaded nanoparticles, the samples were initially in liquid form, placed as a droplet on the diamond surface and measured after water was removed through evaporation using a gentle nitrogen flow.

#### 2.7.4. Dynamic Light Scattering (DLS)

DLS measurements were conducted using an ALV/CGS-3 Compact Goniometer System (ALV GmbH, Langen, Germany) equipped with a 22 mW He–Ne laser (wavelength 632.8 nm) and an ALV-5000/EPP multi-tau correlator with 288 channels. The system also featured an ALV/LSE-5003 module for precise goniometer control and a Polyscience model 9102 bath circulator to regulate the temperature of the measuring cell. All samples were filtered through 0.45 μm hydrophilic PVDF filters prior to analysis. The scattered light intensity and autocorrelation functions were recorded at a fixed angle of 90°, averaging three measurements per sample. Data were processed using the cumulants method and the CONTIN algorithm, which employs the Stokes–Einstein relationship to determine the hydrodynamic radius (R_h_) and size distribution. Measurements at various temperatures included a 15-min equilibration period between temperature changes, which was enough for temperature stabilization.

#### 2.7.5. Fluorescence Spectroscopy (FS)

FS was utilized to determine the critical aggregation concentration (CAC) of the hyperbranched copolymers and to study the encapsulation behavior of curcumin-loaded nanoparticles. The measurements were performed using a NanoLog Fluorimeter (Horiba Jobin Yvon, Kyoto, Japan) equipped with a NanoLED laser diode excitation source (440 nm, 100 ps pulse width) and a UV TBX-PMT series detector (250–850 nm). Hyperbranched copolymer solutions were prepared by successive dilution of a stock solution to achieve a concentration range of 10^−3^–10^−8^ g/mL. A pyrene solution in acetone (1 μL/mL, pyrene concentration 3 × 10^−7^ M) was added to each vial, and the samples were equilibrated for 24 h to allow encapsulation of pyrene into the hydrophobic domains of the polymer aggregates and evaporation of acetone. The fluorescence spectra were recorded with an excitation wavelength of 335 nm, and emission spectra were collected in the range of 355–640 nm. The hydrophobicity of the pyrene environment was assessed by analyzing the I_1_/I_3_ ratio, which represents the ratio of the intensities of the first and third vibronic peaks in the pyrene fluorescence spectra. For curcumin-loaded particles, the same instrument was used with a curcumin excitation wavelength of 430 nm, and emission spectra were recorded over the range of 450–735 nm.

#### 2.7.6. UV–Vis Spectroscopy

UV–Vis spectroscopy was performed using a Perkin-Elmer Lambda 19 spectrophotometer (Waltham, MA, USA). The measurements were conducted using quartz cuvettes.

#### 2.7.7. Electrophoretic Light Scattering (ζ-Potential)

Zeta potential measurements were performed to evaluate the surface charge of polymer particles in solution. The experiments were conducted using a Nano Zeta Sizer instrument (Malvern Instruments Ltd., Malvern, UK) equipped with a 4 mW He–Ne laser operating at a wavelength of 633 nm and measuring scattered radiation at a backscattering angle of 173°. The ζ-potential values were determined using laser Doppler velocimetry (LDV) and analyzed through the Smoluchowski equation. Each reported ζ-potential value represents the average of 50 repeated measurements.

## 3. Results

### 3.1. Synthesis of P(DMAEMA-co-BzMA) Hyperbranched Copolymers

**Scheme 1:**
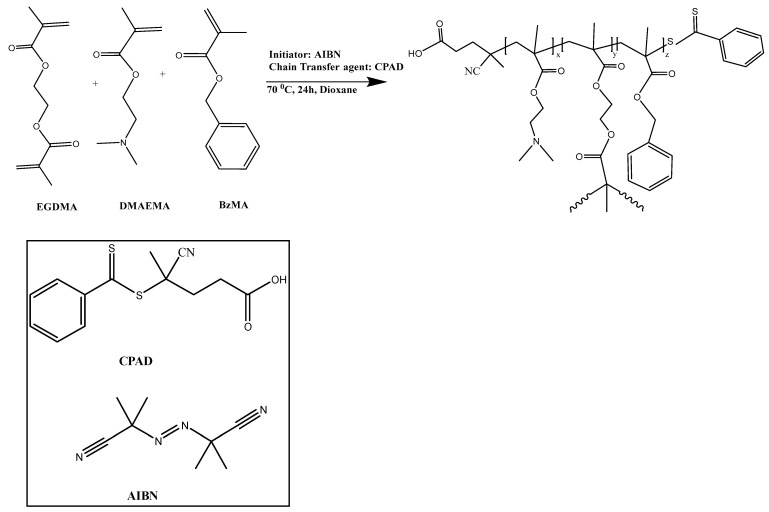
Reaction scheme of the synthesis of P(DMAEMA-co-BzMA) hyperbranched copolymers.

Using RAFT polymerization techniques, two P(DMAEMA-co-BzMA) hyperbranched copolymers with distinct chemical compositions were synthesized. Throughout all experiments, the EGDMA/CTA ratio was maintained at 1.2. AIBN served as the radical initiator. P(DMAEMA-co-BzMA) was synthesized over the course of 24 h at 70 °C (Scheme 1). CPAD was chosen as the CTA agent because of its effectiveness in RAFT polymerization of methacrylic monomers [20], while EGDMA was used as the branching agent. The anticipated chemical structure of the hyperbranched copolymers were analyzed using ^1^H-NMR spectroscopy and FT-IR spectroscopy (Figure S1).

### 3.2. ^1^H-NMR Analysis

**Figure 1:**
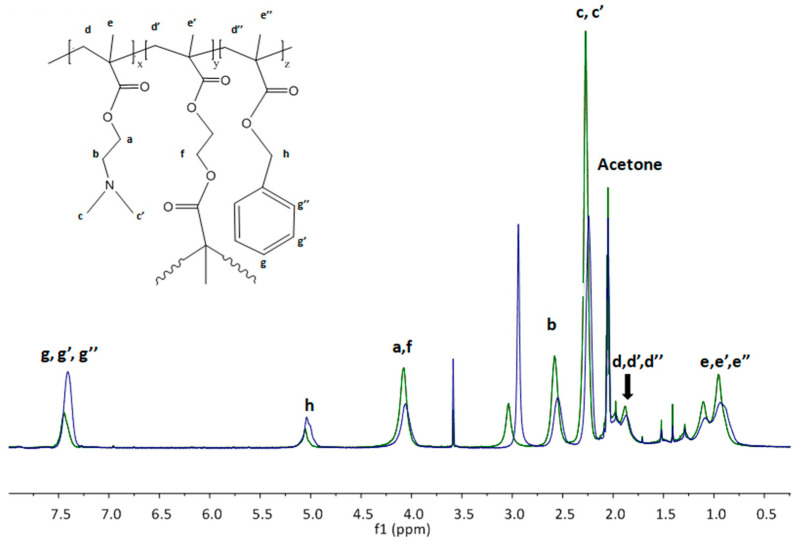
^1^H-NMR spectra of HB1 (green) and HB2 (blue) hyperbranched copolymers (the solvent is deuterated acetone (CD_3_)_2_CO, and the letters indicate the corresponding hydrogens in the chemical structure).

The chemical structure and composition of P(DMAEMA-co-BzMA) hyperbranched copolymers were determined by ^1^H-NMR spectroscopy. The peaks exhibited differences in intensity and width in each spectrum, determined by the composition of the copolymers, as shown in Figure 1. The successful synthesis is confirmed by characteristic peaks, such as those of -CH_3_- groups corresponding to the amino group of the PDMAEMA moiety (peak c,c’) and appearing at 2.27 ppm, as well as those of -CH- groups of an aromatic ring(peak g,g’,g’’) that appear at 7.44 ppm and prove the presence of benzyl methacrylate segments. The same characteristic peaks were selected to calculate the composition of the copolymers. It is important to mention that it is not possible to calculate the exact composition of BzMA due to the overlap of the -CH- groups on the aromatic ring of the CTA (CPAD) at 7.44 ppm, but this contribution is considered to be small.

### 3.3. SEC Analysis

**Figure 2:**
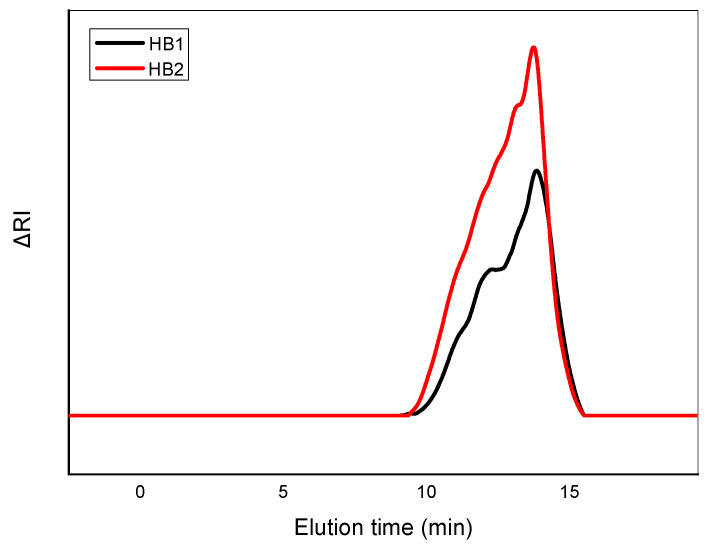
SEC chromatogram for HB1 and HB2 hyperbranched copolymers.

Figure 2 presents the SEC traces for the P(DMAEMA-co-BzMA) copolymers. The molecular weights (M_w_) of each copolymer were 23,300 g/mol and 29,500 g/mol, with polydispersity indices (Ð) of 3.37 and 3.38, respectively (Table 1). It is important to note that the molecular weights obtained by SEC are apparent values, as the hyperbranched methacrylate-based copolymers have different hydrodynamic properties compared to the linear polymer standards used for calibration. Unfortunately, our SEC setup does not have LALS-RALS-viscometer detectors. In any case, due to the hyperbranched and statistical nature of the copolymers, as well as their inherent compositional heterogeneity and the fact that the two comonomers have different refractive indices, the precise determination of the true molar mass is not an easy task—only the size distribution of the copolymers can be assessed.

### 3.4. Self-Assembly in Aqueous Media

**Figure 3:**
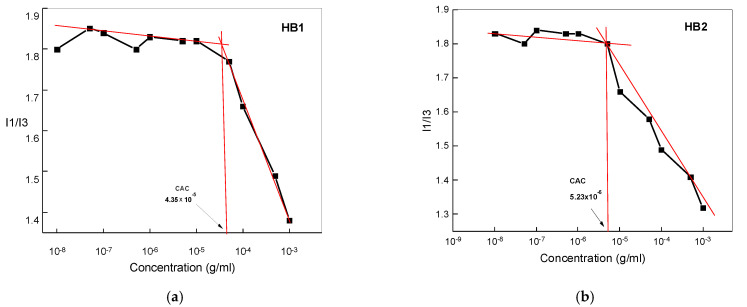
Critical aggregation concentration (CAC) determination for HB1 (**a**) and HB2 (**b**) at pH 7.

Studies on the self-assembly of the hyperbranched copolymers were conducted using various physicochemical techniques. In this regard, the aggregation properties of the copolymers were investigated to determine the critical aggregation concentration (CAC), which represents the concentration threshold where polymer chains begin to form aggregates. Fluorescence spectroscopy was employed as the characterization method, utilizing pyrene—a hydrophobic fluorescent probe known for its sensitivity to environmental polarity. Pyrene tends to be localized within hydrophobic regions of polymer assemblies, making it an effective tool for examining the self-organization behavior of polymeric systems in solutions. To identify the CAC, the intensity ratio between the first (I_1_) and third (I_3_) peaks of the pyrene emission spectrum was measured across a range of polymer concentrations (10^−8^ to 10^−3^ g/mL). The I_1_/I_3_ ratio serves as an indicator of the local polarity; lower values suggest a hydrophobic microenvironment, whereas higher values point to a more hydrophilic system. The values obtained for the hyperbranched copolymers at various pH levels are presented in Table S1. Solutions of the copolymers were prepared in different concentrations and fluorescence measurements were performed to determine the point at which aggregation initiates. The analysis involved plotting the I_1_/I_3_ ratio as a function of polymer concentration. The CAC was identified at the intersection point of two distinct tangents in the plotted data, where a sharp change in the I_1_/I_3_ ratio indicated the formation of polymer aggregates. Figure 3 illustrates the plotted data for I_1_/I_3_vs the concentration of hyperbranched copolymers, from the pyrene fluorescence spectra, at the initial pH (~ pH 7). In both instances, a distinct plateau is observed at low concentrations, indicating the absence of aggregates, while as the concentration increases, the formation of aggregates begins to be observed. As demonstrated in Figure 3, the chemical composition of the copolymers influences the CAC, with copolymer HB2 exhibiting a lower CAC, a reasonable outcome due to the higher hydrophobic BzMA and the lower hydrophilic DMAEMA content of the copolymer. It is also important to note that DMAEMA segments undergo partial deprotonation at pH 7, resulting in a transition to a relatively hydrophilic state.

**Figure 4:**
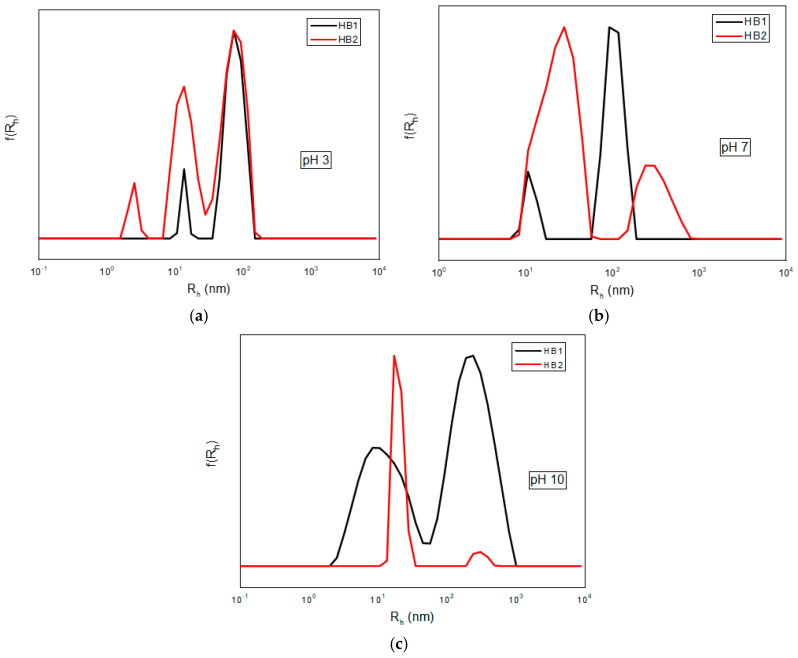
Size distributions from DLS for the HB1 and HB2 hyperbranched copolymer solutions at (**a**) pH 3, (**b**) pH 7, and (**c**) pH 10.

DLS analysis was conducted to investigate the self-assembly behavior of P(DMAEMA-co-BzMA) hyperbranched copolymers, focusing on the determination of the hydrodynamic radius (R_h_) and size polydispersity index (PDI). The scattering intensity of light in DLS measurements is directly related to the mass of the formed nanoparticles, which provides information about the aggregation states of the polymers under varying conditions. Specifically, pH and temperature changes were analyzed to observe their impact on the self-organization of the copolymers. The size distributions of aggregates for the HB1 and HB2 hyperbranched copolymers across three different pH values are illustrated in Figure 4. For the HB1 copolymer, the presence of two populations with distinct sizes is observed (also for HB2, see below). This should be related to the inherent molar mass and compositional and structural heterogeneity of the copolymers as it can also be discerned by SEC analysis (Figure 2). The heterogeneity in macromolecular chain mass, monomer composition, and branching mode of these amphiphilic copolymers most probably leads to the formation of diverse aggregates with different structural characteristics in water which may be observed at different pHs. At neutral pH, the amino groups of PDMAEMA are partially protonated. As the pH decreases, these amino groups become fully protonated. Consequently, repulsive forces are generated within the polymer chains, which break apart the aggregates, leading to a reduction in the mass and size of the supramolecular structures formed in solution. Conversely, at pH 10, the amino groups of PDMAEMA are deprotonated, resulting in a more hydrophobic character for the copolymer system. The chains cluster more and this leads to the formation of larger aggregates and, consequently, an increase in hydrodynamic radius. For the HB2 copolymer, two populations are also observed at neutral pH, but with notable distinctions. The main population shows a broader size distribution and a smaller hydrodynamic radius compared to HB1, while the minor population appears to form larger aggregates. This behavior is likely due to the composition of HB2, which contains a higher proportion of BzMA. The hydrophobic nature of BzMA tends to cause aggregation, leading to a wide range of particle sizes in the solution and the formation of larger aggregates. At pH = 3, the large aggregates break down due to the protonation of DMAEMA amino groups, and also the presence of single chains (R_h_~3 nm) is observed, because of the increased hydrophilicity of the system.At pH=10, a decrease in the hydrodynamic radius of HB2 is noted, which might initially seem unexpected. However, the parallel significant increase in scattering intensity suggests the presence of highly compact aggregates. This is anticipated due to the deprotonation of DMAEMA in combination with the hydrophobic nature of BzMA.

**Figure 5:**
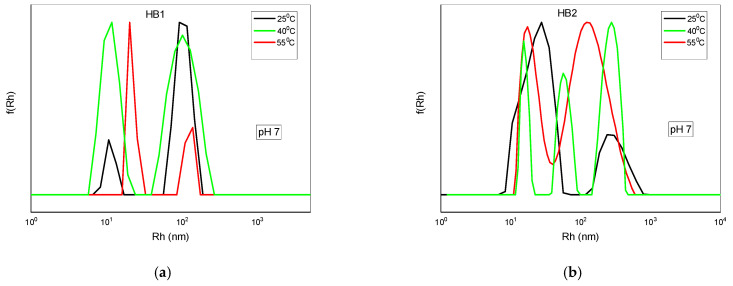
Size distributions from DLS for the HB1 (**a**) and HB2 (**b**) hyperbranched copolymer solutions as a function of temperature.

Due to the thermoresponsive nature of DMAEMA, which exhibits a lower critical solution temperature (LCST) in water between 40 °C and 50 °C, the DLS measurements were conducted as a function of temperature to investigate its effect on the self-assembly of the HB1 and HB2 hyperbranched copolymers. Measurements were conducted at pH 7, and across a temperature range of 25 °C to 55 °C; the results are shown in Figure 5. It is observed that for the HB1 copolymer, at 40 °C, which is close to body temperature, the hydrodynamic radius of the larger populations have not changed compared to those at 25 °C; however, the population with the smaller radius has increased. As the temperature rises to 55 °C, the primary population shifts to the left. This shift occurs because DMAEMA transitions to a hydrophobic state at temperatures above the LCST, prompting aggregation within the system. This behavior is due to temperature-induced changes in intermolecular interactions, leading to the breakdown of hydrogen bonds between water and DMAEMA monomeric units, thus increasing copolymer hydrophobicity. For HB2, there is no significant shift of populations toward a smaller radius; instead, larger aggregates are formed. This is a result of the higher content of hydrophobic BzMA and the lower content of thermoresponsive DMAEMA segments. A summary of the measurements on the self-assembly behavior of the HB1 and HB2 copolymers in aqueous solution across various pHs and temperatures is provided in Table 2. For both copolymers, smaller particles are observed at pH 3, where the amino groups of DMAEMA are fully protonated, accompanied by a low intensity for HB2. At 55 °C, a significant increase in intensity is observed compared to 25 °C at pH 7, although this change is not accompanied by an increase in particle radius. This observation suggests that the aggregates become more compact at 55 °C. ζ-potential measurements were also conducted to evaluate the surface charge and colloidal stability of the hyperbranched copolymers. As expected, positive ζ-potential values were recorded at pH 3 and pH 7, which are attributed to the full and partial protonation of the DMAEMA amino groups, respectively. In acidic conditions (pH 3), PDMAEMA is protonated and exists in a cationic form, aligning with the positive ζ-potential values observed for both HB1 and HB2 copolymers, as shown in Table 2. Conversely, at pH 10, where full deprotonation of the amino groups occurs, negative ζ-potential values were obtained. This negative charge is likely due to the presence of COOH groups originating from the chain transfer agent fragments attached to the macromolecules. These carboxyl groups are exposed on the particle surface due to the random structure of the copolymer and are deprotonated at alkaline pH, resulting in negatively charged COO^–^ groups. Additionally, OH^–^ion adsorption on the particle surface may further contribute to the negative ζ-potential of the particles observed at pH 10.

### 3.5. Effect of Ionic Strength on P(DMAEMA-co-BzMA) Solution Assembly

**Figure 6:**
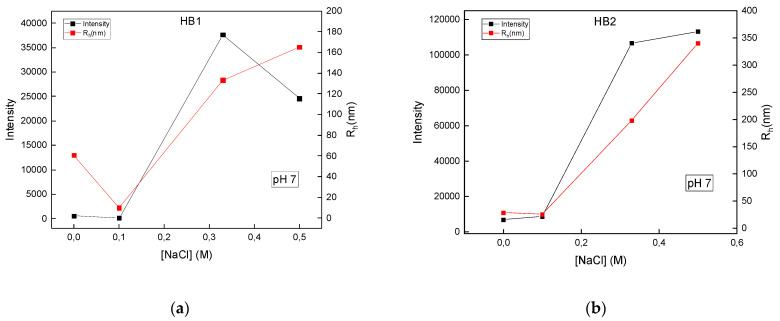
Ionic strength dependence plots for (**a**) HB1 and (**b**) HB2 copolymers (polymer concentration 10^−3^ g/mL, at pH = 7 and at a temperature of 25 °C).

DLS measurements were also performed to assess the impact of ionic strength on the self-organization of the copolymers. For the copolymer HB1 at pH 7, a substantial increase in scattering intensity is observed, accompanied by an increase in hydrodynamic radius with the addition of salt, up to a NaCl concentration of 0.33 M. This result aligns with expectations, as salt ions shield the charges of the partially protonated DMAEMA groups, facilitating copolymer chain aggregation. However, with the final addition of salt at 0.5 M, a reduction in scattering intensity and an increase in hydrodynamic radius were recorded. This phenomenon is likely due to enhanced electrostatic interactions between polymer chains at higher salt concentrations, which induce system relaxation, causing a decrease in aggregate mass as solvent molecules penetrate the aggregates (swelling). For the HB2 copolymer, an even more pronounced increase in both scattering intensity and hydrodynamic radius is observed. This result is expected, given the higher proportion of the hydrophobic component in HB2 compared to HB1, which favors aggregation. The results are shown in Figure 6. The same experiment was conducted at pH 3; however, no significant changes in scattering intensity or hydrodynamic radius were observed. This is likely due to the fully protonated amino groups of DMAEMA at pH 3, leading to strong repulsive forces between chains that prevent particle aggregation upon salt addition.

### 3.6. Curcumin Encapsulation in P(DMAEMA-co-BzMA) Aggregates

Curcumin (CUR), a model hydrophobic drug, was used to determine the drug-carrying potential of the P(DMAEMA-co-BzMA) hyperbranched copolymers with two different compositions. The mixed copolymer/drug nanoparticles were analyzed using DLS, ELS (Table S2), and spectroscopy techniques, including fluorescence, UV–Vis, and FT-IR spectroscopy (Figure S2). Additionally, the encapsulation efficiency of the CUR-loaded copolymers was evaluated (Table S3). For both the HB1 and HB2 hyperbranched copolymers, solutions were prepared with targeted curcumin encapsulation levels of 10% and 20% *w*/*w*. The final concentration of these copolymer solutions was set to 1 × 10^−3^ g/mL after solvent evaporation. Drug-loaded assemblies were observed at 25 °C and a 90° angle using DLS to study size distribution changes following CUR encapsulation. UV–Vis spectroscopy confirmed the successful incorporation of curcumin into the polymeric aggregates, with absorption signals indicating encapsulation. Notably, visible observation further verified encapsulation success, as the curcumin-loaded solutions exhibited a dark orange color without any precipitation, indicative of stable nanoparticle formation. The DLS results for the P(DMAEMA-co-BzMA) copolymer nanoparticles, before and after CUR loading, are shown in Table 3. In both copolymers, encapsulation of curcumin induces observable alterations in their self-organization. For the HB1 copolymer, a substantial increase in intensity—and consequently in the nanoparticle mass—is observed for both the 10% *w*/*w* and 20% *w*/*w* curcumin systems, suggesting the formation of mixed polymer–curcumin particles. Conversely, in the HB2 copolymer, no significant change in intensity is detected between the pre- and post-encapsulation states for either curcumin concentration (10% or 20% *w*/*w*). However, a reduction in the hydrodynamic radius is observed following curcumin encapsulation. This outcome may be influenced by the polymer composition, as HB2 contains a higher proportion of the hydrophobic BzMA component compared to HB1. Therefore, the formation of smaller particles upon the addition of hydrophobic curcumin may be attributed to enhanced hydrophobic interactions within the mixed nanoparticles.

After encapsulating curcumin (CUR) within hyperbranched copolymer aggregates, DLS measurements were conducted on the systems, with measurements taken at 25 °C from day one to day twelve following preparation, at intervals of two days, to assess their stability over time (Figure S3). Overall, the findings indicate a relatively high level of stability over the observation period.

**Figure 7:**
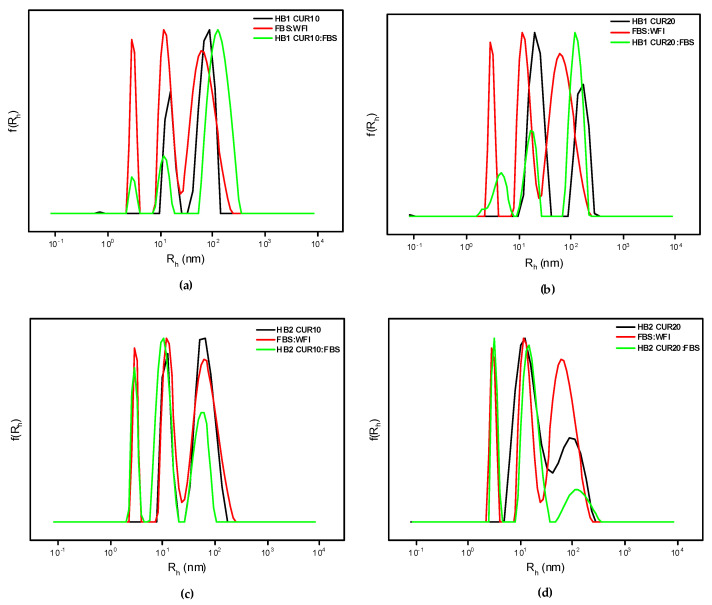
Comparative size distributions of HB1 and HB2 copolymers with curcumin encapsulationat 10%*w*/*w* (**a**,**c**) and 20% *w*/*w* (**b**,**d**) in different media.

Subsequently, the interactions of CUR-loaded nanoparticles with FBS proteins were investigated to assess their potential biomedical application and compatibility with blood components. FBS served as a blood-mimicking medium in the DLS measurements. These measurements assessed nanoparticle characteristics after introducing CUR-loaded nanoparticles to the FBS/WFI solution, as detailed in Section 2.6. Fetal bovine serum (FBS) typically displays three distinct peaks in size distributions from CONTIN analysis, as illustrated in Figure 7. The DLS data shown in this figure indicate that the CUR-loaded nanoparticles do not experience any further aggregation of the previously formed mixed copolymer–drug particles after being combined with FBS/WFI media. This suggests that at a 1:1 ratio of FBS to WFI, the mixed nanostructures do not interact with the components of FBS, allowing the populations of the loaded aggregates to remain unchanged. Therefore, the CUR-loaded copolymer aggregates demonstrate significant stability in the presence of serum proteins. This observation is particularly interesting, as FBS contains albumin, a negatively charged protein, which would be expected to interact with the cationic groups of the polymers. The fact that this interaction does not occur is likely attributed to the structure of the polymer aggregates, which hinders such interactions.

**Figure 8:**
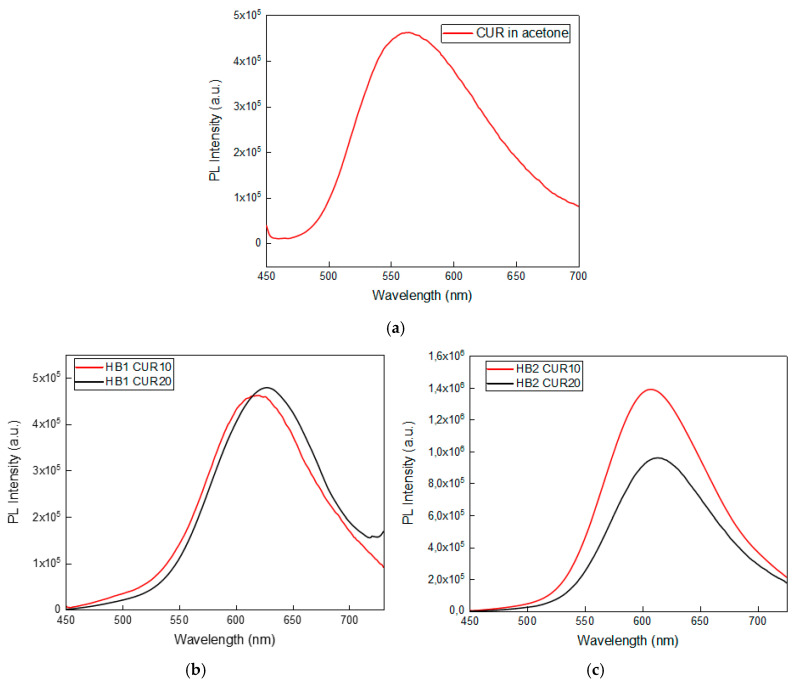
Fluorescence of curcumin in acetone (**a**) and of HB1 aggregates at 10%*w*/*w* and 20%*w*/*w* curcumin encapsulation (**b**) and HB2 ones at 10%*w*/*w* and 20%*w/w* curcumin encapsulation (**c**).

Curcumin is widely recognized for its strong intrinsic fluorescence, making it valuable for bioimaging applications, a property that has led to extensive studies on curcumin’s optical properties [21]. Curcumin exhibits limited water solubility (4.2 μg/mL) [22], which improves drastically when encapsulated in polymeric structures. Moreover, the loaded polymeric aggregates exhibit significant fluorescence after CUR entrapment. Hence, fluorescence studies of CUR-loaded hyperbranched copolymers were performed at pH 7 using UV–Vis and fluorescence techniques, to determine the optical properties of curcumin after its entrapment into the polymeric aggregates. UV–Vis studies are depicted in Figure S4. According to the literature, curcumin in aprotic solvents such as acetone gives a fluorescent signal at 494 to 538 nm [23]. The peak maximum of curcumin in Figure 8a was measured at 562 nm. However, in the studied systems, curcumin shows a signal at higher wavelengths, between 607 and 619 nm (Figure 8b,c). This shift is attributed to the aggregation of curcumin molecules within the hydrophobic regions of the copolymer aggregates [24]. This explains the signal at higher wavelengths observed in HB2 compared to HB1, as HB2 contains a greater amount of BzMA, and therefore has more hydrophobic regions. Moreover, the phenyl rings of BzMA are expected to interact favorably with curcumin molecules, promoting its encapsulation and probably a different way of its aggregation within the copolymer nanostructures.

Curcumin molecules tend to cluster within hydrophobic regions, leading to the formation of dimers and interactions with polymer chains. This clustering lowers the π–π* energy gap between curcumin’s molecular orbitals. Additionally, the dimethylamino groups in DMAEMA can engage in acid–base interactions with the phenolic hydroxyls of curcumin, potentially ionizing these groups and enabling additional interactions beyond simple hydrogen bonding within the hydrophobic core [12,25]. Consequently, curcumin is likely dispersed throughout the polymer matrix, rather than being solely confined to the polymer’s hydrophobic regions, due to its interactions with DMAEMA’s more hydrophilic segments. This distribution results in drug-loaded particles that absorb at higher wavelengths, as curcumin is not fully dissolved within the nanoparticle’s hydrophobic domains.

## 4. Conclusions

The synthesis of amphiphilic, multi-responsive hyperbranched copolymers, P(DMAEMA-co-BzMA), with two different compositions of the hydrophilic, pH- and thermoresponsive DMAEMA component and the hydrophobic BzMA component, was achieved via RAFT polymerization. The successful synthesis was supported by ^1^H-NMR spectroscopy. Molecular weights and molecular weight distributions were confirmed using size exclusion chromatography (SEC). Comprehensive studies of the self-assembly behavior of the hyperbranched copolymers in aqueous solutions were conducted using dynamic light scattering (DLS), electrophoretic light scattering (ELS), and fluorescence spectroscopy (FS). The results demonstrated that the copolymers exhibit stimuli-responsive behavior, due to their sensitivity to changes in pH, temperature, and ionic strength. At pH 3, where DMAEMA amino groups are fully protonated, the copolymers form smaller aggregates. In contrast, when partial protonation or total deprotonation of the amino groups takes place (at pH 7 and pH 10, respectively), larger aggregates or nanoparticles of higher mass appeared, with aggregate size decreasing and mass increasing as the temperature rises at pH 7. The ionic strength of the solution also influences the structural behavior of the copolymers in aqueous media at pH 7, with higher NaCl concentrations leading to the formation of larger aggregates. The CUR-loaded polymeric aggregates demonstrate notable colloidal stability for up to 12 days. In the presence of fetal bovine serum (FBS), the CUR-loaded aggregates exhibited no significant interactions with serum proteins.The ability of the copolymers to encapsulate curcumin was qualitatively observed through the color of the curcumin-loaded polymer solutions as well as their UV–Vis spectra. This was further supported by FT-IR analysis, where new peaks appeared in the spectra of the curcumin-loaded polymers, indicating the interactions between the copolymers and curcumin. Furthermore, the encapsulation efficiency (%) of the polymers was evaluated, with the HB1 polymer, in particular, demonstrating more satisfactory results. Additionally, the CUR-loaded nanoparticles exhibit strong fluorescence, suggesting their applicability in bioimaging. These results underscore the potential of P(DMAEMA-co-BzMA) hyperbranched copolymers as versatile platforms for stimuli-responsive drug delivery and bioimaging applications.

## Figures and Tables

**Table 1:** Characteristics of the P(DMAEMA-co-BzMA) hyperbranched copolymers.

Sample	M_w_ (g/mol) [a]	M_w_/M_n_ [a]	%wt DMAEMA [b]	%wtBzMA [b]
HB1	23,300	3.37	83	17
HB2	29,500	3.38	60	40

[a]: determined by SEC. [b]: determined by ^1^H-NMR.

**Table 2:** Structural characteristics of HB1 and HB2 hyperbranched copolymers in aqueous media.

Sample	T (°C)	pH	Intensity (Kcps)	R_h_ (nm)	PDI	ζp (mV)
HB1	25	3	479	13/74	0.39	+62.6
		7	554	11/104	0.51	+32.7
		10	322	240/11	0.51	−37.1
	40	7	515	11/102	0.49	-
	55	7	5760	20/125	0.32	-
HB2	25	3	173	2/72/14	0.49	+68.5
		7	6720	23/301	0.43	+40.5
		10	5270	20/300	0.14	−47.0
	40	7	21580	16/58/267	0.49	-
	55	7	20226	17/123	0.49	-

**Table 3:** DLS data before and after curcumin encapsulation in HB1 and HB2 hyperbranched copolymer aggregates.

Sample	% CUR	Intensity Without/With CUR	R_h_ (nm) Without/With CUR	PDI Without/With CUR
HB1	10%	554/6160	11 and 104/15 and 75	0.52/0.43
	20%	554/15968	11 and 104/21 and 160	0.52/0.45
HB2	10%	6720/6960	23 and 301/12 and 65	0.43/0.48
	20%	6720/4730	23 and 301/12 and 85	0.43/0.42

## Data Availability

The original contributions presented in this study are included in the article/Supplementary Material. Further inquiries can be directed to the corresponding author.

## Supplementary Materials

The following supporting information can be downloaded at https://www.mdpi.com/article/10.3390/ma18030513/s1:Table S1. I_1_/I_3_ measurements of HP(DMAEMA-co-BzMA) hyperbranched copolymers at different pH values; Figure S1. ATR-FTIR spectrum of HB1 and HB2 hyperbranched copolymers; Table S2. Zeta potential measurements of CUR-loaded P(DMAEMA-co-BzMA) hyperbranched copolymers at pH 7 and 25 °C; Figure S2.FTIR spectra of curcumin (a) and CUR-loaded HB1 copolymer at 10% *w*/*w* curcumin encapsulation (upper spectrum) and HB1 copolymer (lower spectrum) (b); Table S3. Curcumin encapsulation in P(DMAEMA-co-BzMA) hyperbranched aggregates; Figure S3. DLS measurements from stability studies for CUR-loaded polymeric nanoparticles of HB1 (a) and (b) and HB2 (c) and (d) copolymers at 10% *w*/*w* and 20% *w*/*w* curcumin encapsulation; Figure S4. UV–Vis spectra from CUR-loaded HB1 (a) and HB2 (b) at 10% *w*/*w* and 20% *w*/*w* curcumin encapsulation.
