# Supplementary material for: Multi-Responsive Amphiphilic Hyperbranched Poly[(2-dimethyl aminoethyl methacrylate)-co-(benzyl methacrylate)]copolymers: Self-Assembly and Curcumin Encapsulation in Aqueous Media
Source: Materials (Basel). 2025 Jan 23;18(3):513. doi: 10.3390/ma18030513 (PMC11818926; doi:10.3390/ma18030513)
Supplement: Supplementary file 1 [file materials-18-00513-s001.zip › materials-3385296-supplementary.pdf]

Article

Multi-Responsive Amphiphilic Hyperbranched Poly[(2-dimethyl aminoethyl methacrylate)-co-(benzyl methacrylate)] copolymers: Self-Assembly and Curcumin Encapsulation in Aqueous Media

Foteini Ginosati, Dimitrios Vagenas, Angelica Maria Gerardos and Stergios Pispas *

Theoretical and Physical Chemistry Institute, National Hellenic Research Foundation, 48 Vassileos Constantinou Ave., 11635 Athens, Greece; fotini.gin97@gmail.com (F.G.); dimitrisv98@gmail.com (D.V.); amgerar@eie.gr (A.M.G.)

* Correspondence: pispas@eie.gr

Table S1. I_1/I_3 measurements of P(DMAEMA-co-BzMA) hyperbranched copolymers at different pH values.

Sample	pH	I_1/I_3
HB1	3	1.66
	7	1.38
	10	1.34
HB2	3	1.44
	7	1.32
	10	-[(a)]

(a) The I_1/I_3 ratio for HB2 copolymer at pH 10 could not be measured due to the formation of a precipitate in the solution. This likely occurred as a result of the strong hydrophobic interactions between BzMAmonomeric units and pyrene.

The results in Table S1 highlight that copolymers with a higher DMAEMA content exhibit an increase in the I_1/I_3 ratio as the pH decreases. This behavior is anticipated, as a greater number of hydrophilic and protonated DMAEMA groups in the copolymer chain create a more distinct hydrophilic microenvironment.

ATR-FTIR spectroscopy of hyperbranched copolymers

FTIR spectroscopy was employed for the qualitative analysis of the copolymers' chemical structure by examining the vibrational peaks associated with the chemical groups present in the hyperbranched copolymers. Figure S1 displays the spectrum of HB2 copolymer. The band observed at 2946 cm^{-1} corresponds to the C-H bond vibrations of the -CH_2 groups from the DMAEMA and BzMA monomers. Peaks at 2821 cm^{-1} and 2770 cm^{-1} are attributed to the bond vibrations of the amine group (-$N(CH_3)_2$) in DMAEMA. The prominent peak at 1722 cm^{-1} corresponds to C=O stretching (ester) of both monomers. The peak at 1455 cm^{-1} corresponds to asymmetric stretching vibrations of the C-H bonds in the –CH_2 groups of both DMAEMA and BzMA. The peak at 1143 cm^{-1} is associated with the vibration of the O=C-O bonds in both DMAEMA and BzMA, though literature suggests it also includes C-N bond stretching vibrations from the diisopropylamino groups in DMAEMA. This overlap of peaks complicates the distinction between these chemical groups. Finally, the peaks at 748 cm^{-1} and 697 cm^{-1} are attributed to monosubstituted benzene rings of the BzMA monomeric units.

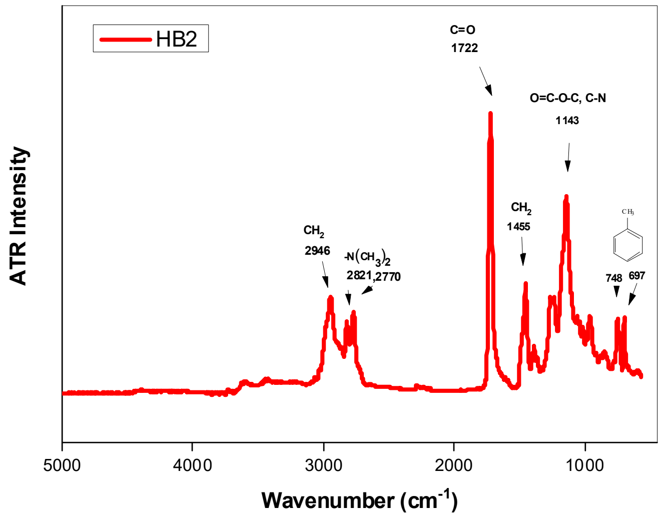

Figure S1. ATR-FTIR spectrum of HB2 hyperbranched copolymer.

Zeta potential measurements of drug-loaded hyperbranched copolymers

Table S2. Zeta potential measurements of CUR-loaded P(DMAEMA-co-BzMA) hyperbranched copolymers at pH 7 and 25 ^{0}C.

Sample	ζp (mV)
HB1 CUR10	+38
HB1 CUR20	+35
HB2 CUR10	+47
HB2 CUR20	+46

The results of the measurements indicate that the drug-loaded copolymers exhibit a slight but not significant increase in their ζ-potential values at pH 7 compared to the empty copolymer aggregates before the encapsulation of curcumin. This possibly suggests that curcumin is primarily encapsulated within the internal part of the polymer aggregates rather than adsorbed on their surface.

ATR-FTIR spectroscopy of drug-loaded hyperbranched copolymers

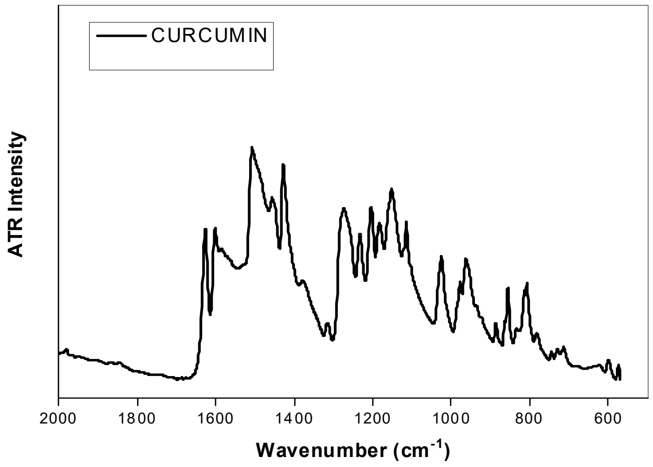

(a)

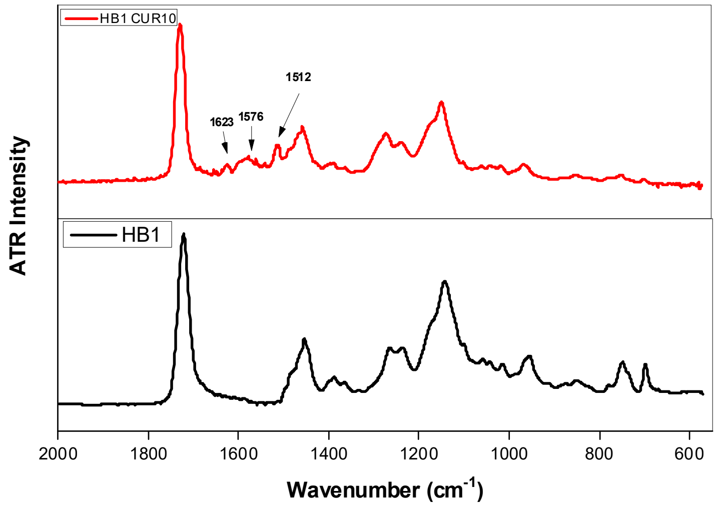

(b)

Figure S2. FTIR spectra of curcumin (a) and CUR-loaded HB1 copolymer at 10%w/w curcumin encapsulation (upper spectrum) and HB1 copolymer (lower spectrum) (b).

Figure S2 presents the FTIR spectra of the HB1 hyperbranched copolymer and the CUR-loaded copolymer. Following curcumin encapsulation, three additional peaks appear. The peak at **1623 cm^{-1}** could be attributed to the **C=O stretching vibration** of the conjugated carbonyl group in the curcumin molecule. The peak at **1512 cm^{-1}** is associated with **C=C stretching vibrations** in aromatic rings, characteristic of the aromatic structure of curcumin. The peak at **1576 cm^{-1}** may correspond to **alkene C=C vibrations** or result from interactions between curcumin and the polymers, such as resonance or hydrogen bonding, which modify the characteristics of the C=C or C=O bonds. Additionally, a shift is observed in the peak attributed to the C-N stretching vibrations of DMAEMA, between the spectrum of the pure copolymer and that of the curcumin-loaded nanoparticles, from 1143 cm^{-1} to 1148 cm^{-1}. Similarly, the peak likely corresponding to the C-H out-of-plane bending vibrations in aromatic groups, shifts from 954 cm^{-1} to 966 cm^{-1}. These changes indicate interactions between curcumin and the copolymer, such as the formation of hydrogen bonds between the hydroxyl groups of curcumin and the amino group of DMAEMA. The FTIR spectra confirm that curcumin has been successfully encapsulated within the copolymer aggregates.

Encapsulation efficiency of drug-loaded hyperbranched copolymers

Table S3. Curcumin encapsulation in P(DMAEMA-co-BzMA) hyperbranched aggregates.

Sample	Quantity of curcumin used (mg)	Maximum encapsulation(%w/w)	% Encapsulation efficiency
HB1 CUR10	1	10	7
HB1 CUR20	2	20	15.4
HB2 CUR10	1	10	4.7
HB2 CUR20	2	20	1.1

It is observed that both copolymers have the ability to encapsulate curcumin, with HB1 demonstrating satisfactory encapsulation efficiency. Although HB2 contains a higher percentage of hydrophobic component its hydrophobic regions seem to be more well-organized and unable to accommodate a significant amount of the hydrophobic drug. Additionally, as indicated by DLS measurements, HB2 exhibits smaller particle sizes both in the pure polymer state and after curcumin encapsulation. In contrast, HB1 forms larger

particles before and after encapsulation, likely due to its larger and more flexible structures, which may provide better accessibilityto its hydrophobic regions for drug loading.

Stability of CUR-loaded hyperbranched copolymers.

The temporal stability of CUR-loaded polymeric nanoparticles of HB1 and HB2 copolymers was evaluated using the DLS technique (at a 90° measuring angle). The results are depicted in Figure S3 as plots showing the scattered intensity and hydrodynamic radius of the loaded aggregates as a function of time.

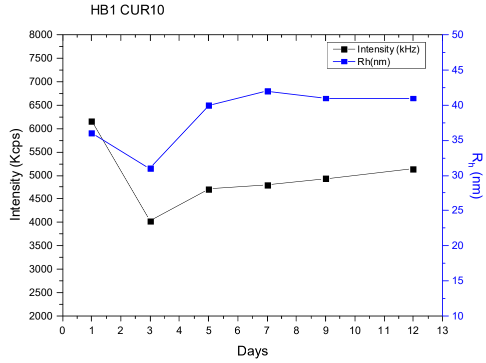

(**a**)

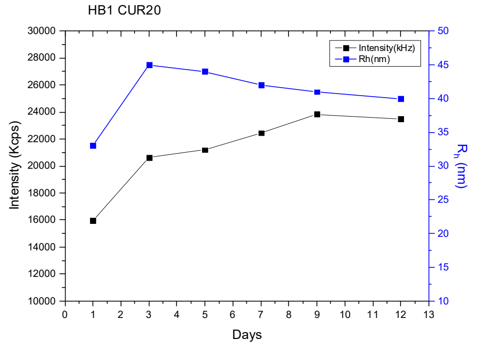

(**b**)

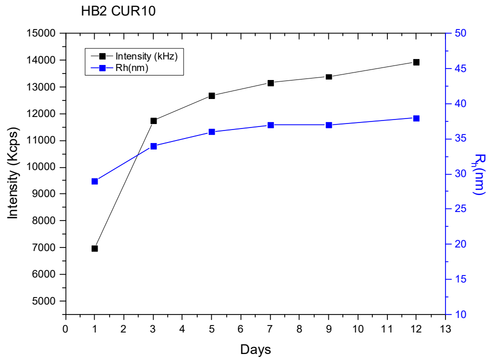

(**c**)

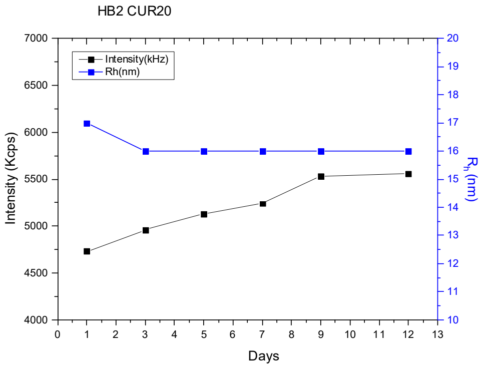

(**d**)

Figure S3. DLS measurementsfrom stability studies for CUR-loaded polymeric nanoparticles of HB1(a) and (b) and HB2 (c) and(d) copolymersat 10%w/w and 20%w/w curcumin encapsulation.

It is noticeable that the HB2 solutions exhibit greater stability over time, as indicated by the measured scattered intensity and hydrodynamic radius. This enhanced colloidal stability can be attributed to the higher BzMA content in the HB2 copolymer, a compound known to stabilize polymeric systems.

UV-Vis spectroscopy of drug-loaded hyperbranched copolymers

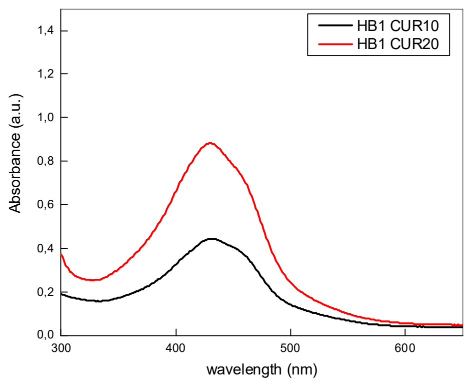

(a)

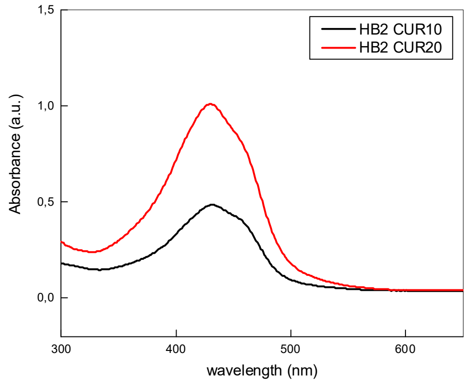

(b)

Figure S4. UV-Vis spectra from CUR-loaded HB1(a) and HB2(b) at 10%w/w and 20%w/w curcumin encapsulation.

The UV-Vis spectra of curcumin-loaded aggregates for the hyperbranched copolymers HB1 and HB2, are illustrated in Figure S4. The characteristic absorption peak of curcumin observed in the aqueous solutions of both copolymers confirms its successful encapsulation.
